# Exoskeletons: A challenge for development

**DOI:** 10.1017/wtc.2022.28

**Published:** 2023-01-05

**Authors:** Klaus Bengler, Christina M. Harbauer, Martin Fleischer

**Affiliations:** Chair of Ergonomics, TUM School of Engineering and Design, Technical University of Munich, Munich, Germany

**Keywords:** Exoskeletons, Control, Design, Biomechanics, Optimisation

## Abstract

The development of exoskeletons is currently a lengthy process full of challenges. We are proposing a framework to accelerate the process and make the resulting exoskeletons more user-centered. The needed accomplishments in science are described in an effort to lay the foundation for future research projects. Since the early 2000s, exoskeletons have been discussed as an emerging technology in industrial, medical, or military applications. Those systems are designed to support people during manual tasks. At first, those systems lacked broad acceptance. Many models found their niches in ongoing developments and more diverse systems entering the market. There are still applications that are in dire need of such assistance. Due to the lack of experience with body-worn robotics, the development of such systems has been shaped by trial and error. The lack of legacy products results in longer development times. In this paper, a process to generate a framework is presented to display the required research to enable future exoskeleton designers. Owing to their proximity to the user’s body, exoskeletons are highly complex systems that need sophisticated subsystems, such as kinematic, control, interaction design, or actuators, to be accepted by users. Due to the wide variety of fields and high user demands, a synchronized multidisciplinary effort is necessary. To achieve this, a process to develop a modular framework for exoskeleton design is proposed. It focuses on user- and use-case-centered solutions for matching kinematics, actuation, and control. To ensure the usefulness of the framework, an evaluation of the incorporated solutions is required.

## 1. Introduction

**Figure 1.**
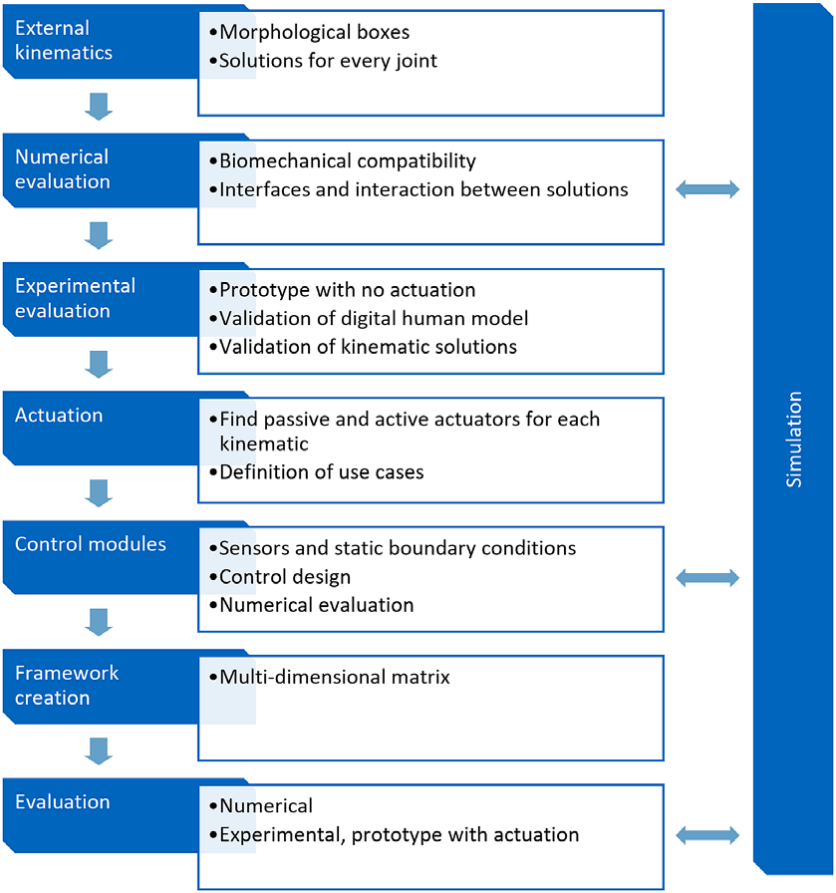
Structure of the development process for the framework as described in Section 3

Exoskeletons are a promising tool to improve physically straining workplaces and contribute to making future work more aging- and age-appropriate. When deployed correctly, exoskeletons have the potential to reduce the load on joints and muscles and thus reduce fatigue during the workday. In the long term, the goal is that these systems will reduce workplace-related musculoskeletal diseases and injuries. Since the first prototypes and market-ready products were developed, many insights have been gained about how this new technology can and should be used. Apart from how it can be integrated into an existing workforce, researchers and manufacturers gained a lot of knowledge on how these systems needed to be designed. And they have not finished learning yet. When exoskeleton design in the past was probably mostly a “trial and error” procedure with frequent product iterations and field studies, now there is potentially enough data to develop a design process for the user-centered design of exoskeletons. However, fundamental research is still being done, particularly in digital human modeling (DHM) with exoskeletons, biomechanically compatible kinematics, actuation, control, and evaluation of exoskeletons. This paper proposes a process to build a framework to find optimal solutions for those fields. Regarding the design aspects, the approach should be free of limitations generated by the current state of the art. Much like the idealized Otto cycle, the framework is an ideal example of how exoskeletons should be designed. Since newly designed exoskeletons need evaluation, evaluation tools will be part of the framework. They will provide the methodology for digital and analog exoskeleton assessment. While these tools will be helpful to design novel exoskeletons, they will also be used to evaluate the framework as described in Section 3.7. In this context, the framework is understood as a reference work with evaluated modular design approaches, where future exoskeletons for new applications can be built upon. The article aims to propose a holistic development framework. The paper describes the needed research. Future exoskeleton developers require these tools and insights from the framework. Varied and detailed aspects of the topics within the process are found in the literature for different use cases, joints, and users. Those need to be combined in a standardized manner since verified scientific knowledge from one exoskeleton type can be transferred to other use cases. While explaining the necessary steps to build such an elaborate framework, a few examples of published research or reviews about those topics from different disciplines are referenced. Some proposed tools are explained in more detail to understand the concepts better. The article’s structure corresponds to the framework’s development process, as shown in Figure 1.

The stages shown in Figure 1 are explained in more detail below and will be expanded in the sections of Section 3.

1. The premise for the standardized development of external kinematics is a simulation toolbox that allows an iterative evaluation in the early stages of the process. Digital human models, elastodynamic models of exoskeletons, and finite-element method (FEM) models need to be developed and harmonized to work in a co-simulation.
2. For each joint of the human body, compatible external kinematics need to be designed in the sense of a morphological box.
3. These kinematics need to be evaluated with standard mechanical and dynamic calculations, as well as with the simulation from 1, for compatibility with human biomechanics and with each other.
4. To evaluate the kinematics and their simulation, an experimental evaluation needs to be conducted with simplistic kinematic prototypes.
5. When it is certain that the kinematics will fit human movements, different existing solutions and new designs for active and passive actuation need to be collected for each possible kinematic.
6. Fitted to each kinematic and active actuators, control modules with respective sensory systems need to be designed and evaluated in simulation settings concerning different possible use cases.
7. The framework must be evaluated. This will be done through an exemplary execution of the framework from a designer’s perspective.
8. All the identified kinematics, actuators, and control modules are combined into a multidimensional solution space matrix and clustered according to use cases.

## 2. State of the art

During the exoskeleton hype, especially for occupational use cases, the challenges and limitations of that rather new technology became clearer, and research projects arose to find solutions. Those solutions were usually directed at very specific problems coming from specific research backgrounds, for example, medical technology, production technology, control engineering, or biomechanics. Reviews regarding recent developments, challenges, and future developments of exoskeletons from different perspectives were published. From 2020 until 2022, 24 review articles relevant to the topics addressed in this article were identified. The authors usually differentiate between the subjects of kinematic design and control strategy, often in a separate manner. For kinematic design challenges like incompatibilities between human and exoskeleton joints, discomfort at the physical human–robot interfaces (pHRIs), and the insufficient mass–power ratio of active exoskeletons are described. Technical details of control strategies are rarely specified in detail. They are discussed rather superficially as a means to increase stability, performance, and achieve natural movements (Gupta et al., 2019, 2020; Chen et al., 2020; Kapsalyamov et al., 2020; Schnieders and Stone, 2020; Pérez Vidal et al., 2021; Plaza et al., 2021; Rodríguez-Fernández et al., 2021; Liang et al., 2022; Massardi et al., 2022; Tijjani et al., 2022). The design of lower limb exoskeletons is a focus topic on its own, especially in rehabilitation and assisted living use cases. In research, the mechanical design (Islam et al., 2017; Viteckova et al., 2018; Del Sanchez-Villamañan et al., 2019; Jamwal et al., 2020; Kermavnar et al., 2021; Rodríguez-Fernández et al., 2021; Zhang et al., 2021; Tijjani et al., 2022) and the suitability of different control strategies for adaptive gait assistance (Jamwal et al., 2020; Baud et al., 2021) is still a major focus. Fewer reviews focus on different control algorithms and sensing (Massardi et al., 2022; Sun et al., 2022), human–machine interface (Giusino et al., 2020) or give a more generalized overview of the topic of exoskeletons (Masia et al., 2018; La Tejera et al., 2021).

Some authors call for more synchronized research between different fields and regulatory institutions (Chen et al., 2020; Fosch-Villaronga and Özcan, 2020; Kapsalyamov et al., 2020). In this regard, there are publications proposing a specific human-centered development process specifically for exoskeletons (Gupta et al., 2020; Monica et al., 2021). Research projects like the one presented by Tröster et al. (2020) and Drees et al. (2021) try to implement development processes that follow an intensive task analysis and a combined exoskeleton mechanics and human modeling approach. The focus is on kinematic and actuator design, but control design is not addressed. The literature analysis of all the different fields involved shows that there is a lot of fundamental research necessary to achieve exoskeletons that are well equipped for their use cases. Furthermore, a more holistic approach is necessary to achieve a human-centered process, since different fields are involved in the mentioned topics like biomechanical compatibility, pHRIs, and natural movement. This is only possible if those fields develop methods together and implement tools that are usable for either side. Relevant identified topics are a simulation of human biomechanics and physiology, kinematic design, actuator design, sensing, control, and the evaluation of all of them.

## 3. Development of the framework

The proposed framework aims to enable future exoskeleton designs optimized for the human and the use case. Depending on the desired supported activity and additional tasks, a range of potential compatible kinematics with suitable actuators, sensors, as well as appropriate control strategies and algorithms can be identified. This shortens the development time by providing a feasible solution space. The following sections describe the development process of the framework.

### 3.1. Simulation

Before designing exoskeletons, sufficient tools must be developed to evaluate such systems digitally to shorten development times and reduce the number of necessary prototypes (Zheng et al., 2021). A co-simulation between a digital human model, elastodynamic simulations, and FEM simulations is proposed. Digital human model software such as OpenSim or Anybody is well established in assessing biomechanical issues in early development stages. It can also be used in the field of exoskeletons. Current biomechanical DHM is multibody simulations with mainly rigid bodies. And even though there are already methodologies to evaluate exoskeletons with these models (Agarwal et al., 2010; Tröster et al., 2020; Fritzsche et al., 2022), those models lack a realistic physical human-exoskeleton interaction. The influence of soft tissues between the exoskeleton and the bones is not well represented in those models.

The multi-body simulation describes two systems: the human body and the exoskeleton. While the human body is modeled with a biomechanical simulation, the exoskeleton is represented by an elastodynamic model. Biomechanical DHM only uses a simplified version of the human body. One example is the elbow joint. Often modeled as a simple hinge joint, the elbow is actually a complex system with changing rotation axis over flexion/extension (Kapandji and Rehart, 2016). Therefore, the developed exoskeleton based on such a DHM where the elbow is assumed as a hinge will have misalignments between the joints of the physical prototype and the human user. This is only one example of how biomechanical DHM needs to be refined to provide a productive input to exoskeleton developers to avoid such misalignments. Researchers and the industry need to generate a biomechanical rigid full-body model with detailed joint characteristics and anthropometric scalability. The exoskeletons are often modeled in an abstracted way inside the environment of the biomechanical DHM. This is, in many cases, a sufficient approach if the exoskeleton is a mainly rigid system. For future developments, however, elastodynamic systems must also be considered to account for the variety and complexity of the human body as opposed to the exoskeleton.

There are different approaches to simulate the physical attachment of exoskeletons onto a DHM (Inose et al., 2017; Kruif et al., 2017; Zhou et al., 2017; Tröster et al., 2018), but they do not account for an unknown pressure distribution or need study data for a realistic representation. Therefore, their suitability for the early stage use is limited to force transfer assessment. While force transfer is an important factor, a tool to test for pressure distribution and thus comfort is advisable. FEM models require much more computational effort than multi-body models but offer more detailed information. The two body regions that can benefit the most from FEM simulations are the attachment points of the exoskeleton onto the user (pHMI) and the human joints. Although the necessity of using FEM for joint tissue simulation has not been determined yet, the pHMI should be assessed using an FEM model. When attaching exoskeletons to that model, the influence of wobbly mass in terms of its relative movement due to elasticity and its changing shape under pressure needs to be represented. This procedure provides necessary insight into the interaction between the exoskeleton and human tissue, as stated in Giusino et al. (2020).

For the simulation of soft tissue behavior in simulation models, Finite-Elements-Method (FEM) simulations are well-established tools. Integrating those into multibody simulation results in the high complexity and massive computing power. Co-simulation between multi-body simulations and FEM models is a beneficial strategy for developing high-fidelity biomechanical models since it makes simultaneous calculation in both tools feasible. In this framework, a co-simulation with the following subsystems and aspects needs to be developed to fit the needs. Thus, two multi-body models – one of the humans, one of the exoskeletons – and an FEM model that simulates the contact surfaces between the human and the exoskeleton, thus connecting the two multi-body models in a co-simulation. The development of new models or co-simulations is time intensive. It is not feasible to develop new ones for every exoskeleton design process. Current models are usually specialized to the purpose of use and only a few generalized models exist. This requires the adoption or generation of new digital human models for each application. Only a limited set of complex and validated models is available due to the considerable effort that needs to be put into their setup. Developers need a compilation of open-source DHM tools for the assessment. Accessibility also needs to be improved so that persons with less experience can operate those complex models. The most promising approach is a cooperation between research and industrial partners to incorporate both parties’ needs and lower the cost of entry for industrial partners.

### 3.2. Catalog of external kinematics

Physical assistance systems such as exoskeletons must be designed with a human-centered design process to achieve maximum acceptance (Schmidtler et al., 2015; Gupta et al., 2020; Monica et al., 2021). This is also true for the kinematics, as exoskeletons impress their kinematics on humans. If the biomechanical compatibility is not secured, several unwanted effects in the physical human-machine interaction occur. Most prominent is the slipping of the attachments induced by shear forces. In human joints, the incompatibility can cause additional forces, thus rendering the system useless or even harmful (Mallat et al., 2019). Good biomechanical compatibility is one preliminary condition of good usefulness of exoskeletons.

To provide a versatile framework, a wide variety of kinematic solutions for each human joint needs to be provided. To achieve high-quality solutions, the human and the exoskeleton joints need to be biomechanically compatible (Pons and José, 2008). In the literature, the kinematics of exoskeletons are often designed to mimic human kinematics, most of the time with simplifications of the latter, such as the elbow having a static center of rotation. Approaches to solving this problem through mathematical solutions can be found in the literature (Jarrasse and Morel, 2011; Cempini et al., 2013; Li et al., 2017). These researchers have shown that analyzing the two serial chains of human and machine can drastically increase the quality of kinematic solutions. Defining these kinematic chains for each human joint and generating exoskeleton joints accordingly leads to a finite number of solutions in abstract topologies. Exoskeletons often directly interact with more than one human joint. Thus, a way of connecting singular kinematics needs to be defined.

In Harbauer et al. (2020), a database was generated in an attempt to collect and cluster all existing exoskeletons’ kinematics. Despite being a good overview, that database alone is not useful for identifying fitting solutions for specific use cases. These kinematics need to be categorized into solution spaces for different applications in the way of morphological analysis (Zwicky, 1967, 1969). Literature-based research is not sufficient because the literature seldom provides a biomechanical evaluation or studies for biomechanical compatibility. Evaluation is highly needed to ensure the quality and usefulness of the identified solution. Future developers need a database of exoskeleton kinematics for all human joints, including categorization and assessment. Application-oriented research projects are required to develop such a database. Experts in the fields of kinematic synthesis and biomechanics are essential to secure the success of such projects.

### 3.3. Numerical evaluation

Sections 3.3 and 3.4 present two steps of evaluation and validation. In the following, the two steps are described as consecutive even though there is a need to coordinate them strictly to be as close as possible to the subjects researched. In digital product design processes, systems must be assessed without prototyping. The combination of the simulation and the kinematic solutions can provide a methodology to evaluate biomechanical compatibility. While evaluating active exoskeletons through DHM has been done already, assessing systems without actuators through simulations is not an established method, even though Jarrasse and Morel (2011) have shown that this approach can lead to improvements.

The evaluation of the not actuated structure generated in Section 3.2 is done with the simulation developed in Section 3.1 using representative movements recorded by a motion-capturing system. Each one of the kinematic solutions and their combinations must be tested against a baseline without external kinematics in several aspects. It is important to note that no actuation of the exoskeleton is intended at this point of the evaluation. When applying the passive external kinematics to the human kinematics, there must be no significant change in the joint reaction forces and no significant shear forces at the attachment points compared to the same motion simulated without the exoskeleton. There is not enough evidential literature to define “significant” or rather harmful changes in this context, thus further research is required. That way, it can be evaluated which solutions are suitable and which are to be dismissed or reworked. Harbauer et al. (2022) described an example of a simulation-based exoskeleton design optimization.

Regarding maximum pressure, which should act on the human body, it depends highly on which limb the exoskeleton is physically connected with the human body and where exactly the pressure on that limb is induced. As reported by Kermavnar et al. (2018), the pain detection threshold of healthy people varies significantly between individuals, between 14 and 34 kPa in the mean for circumferential tissue compression. Regarding maximum pressure, which should act on the human body, it depends highly on which limb the exoskeleton is physically connected with the human body and where exactly the pressure on that limb is induced.

Pressure sensitivity also varies significantly within a single person. For different points on the human back and shoulders, Fischer (1987) reported values with means ranging in the mean from 3.7 kg/cm² at the upper trapezius up to 9.0 kg/cm² at the paraspinal for men, with significant differences between men and women. Since those are mean values, those would not be acceptable for 50% of the population and, therefore, unsuitable for exoskeleton design. In Fischer (1987), the 99.5th percentile values are 0.8 kg/cm² (upper trapezius) and therefore the safest for exoskeleton design. With the pressure values between the human and the exoskeleton identified in the biomechanical modeling and with the safe values for discomfort in mind, either the kinematics can be optimized, or the surfaces of the attachment points need to be designed in a way that they distribute the forces safely and thus reduce the local pressure. Within the framework, this numerical evaluation is step two for future developers. The open-source simulation and the chosen kinematics from the first step are needed. The result is an optimized exoskeleton concept that they can prototype and transfer to the next step: the experimental evaluation.

### 3.4. Experimental evaluation of simulation models and external kinematics

To validate the simulations proposed in Section 3.3, a participant study with selected exoskeletons without actuation needs to be conducted. A selection of the identified suitable kinematics is prototyped as accurately to the theoretical structures as possible. The representative movements recorded in Section 3.3 are reproduced by the participants. In the numerical evaluation, joint reaction forces and shear forces at the attachment points are used as characteristics for good biomechanical compatibility. Ideally, a participant study that correlates the joint reaction forces, as well as pressure and shear forces at the attachment points to the subjective discomfort of the participants, is conducted. This way, the simulation of joint reaction forces as well as the pressure and shear forces at the attachment points can be validated. Additionally, the quality of the built kinematic solutions can be evaluated and compared to the simulation results. Measurement of the pressure at attachment points is a well-researched topic. Existing methodologies and tools are applicable in living subject studies with little concern from an ethical point of view (Kermavnar et al., 2018). On the contrary, measuring joint reaction forces poses a greater challenge since it is necessary to implant sensors in the joints of alive participants or at least in cadaver studies (D’Lima et al., 2012). The joint reaction forces may thus not be available in the validation process and may need to be replaced by complementary methods such as classical analysis methods (Reilly and Martens, 1972), biomechanical assessments through measurement and simulation, or neural networks enhanced EMG (Vries et al., 2016). The central aspect of Sections 3.3 and 3.4 is the quality of the kinematic solutions generated in Section 3.2. Additionally, the proposed numerical evaluation allows the evaluation of the methodology through which the kinematic solutions were developed (Section 3.2), the simulation tool (Section 3.1), and the quality of the prototype itself. This is a chicken-or-egg problem since the simulation is needed to generate the catalog and the solutions from the catalog are needed to validate the simulation through participant studies. An iterative procedure might be necessary depending on the results of the validation of the simulation. The human-exoskeleton kinematic chain can now be designed with high biomechanical compatibility. It will not hinder any movements and the attachment points will not move relatively to the users’ contact surfaces. The next step is then to find feasible actuators to enable assistance through the exoskeleton.

### 3.5. Catalog of actuation

The actuation of an exoskeleton is commonly separated into passive and active actuators. While passive actuators (e.g., springs) absorb energy from users and recuperate in beneficial moments to redistribute the stress throughout the movement, active actuators (e.g., linear actuators) draw energy from an arbitrary energy source or storage (e.g., battery) to insert them into the system. Kinematic solutions need to have actuation, be it passive or active. Similar to the catalog of kinematics, a catalog of fitting actuation methods will help designers to make good choices for their use case. Therefore, a cluster of actuators needs to be developed for implementation in this framework. To achieve this, essential properties are suitable, such as:

1. Range of motion
2. Speed
3. Power in- and output
4. Direction of motion (e.g., linear, rotating…)
5. Force/torque output
6. Active/passive
7. …

The kinematics and the representative motions generate the requirements for optimal actuators in each use case. In this analysis, it is likely not relevant whether the actuator is active or passive. To proceed further, an assessment of the currently available actuators and their fitting to the requirements is instrumental. Developing new actuators following the requirements might be inevitable to succeed in future projects. Whether a system should be active, passive, or hybrid for a particular use case is to be determined. Current research suggests that passive exoskeletons are more beneficial in static use cases since they show even lower EMG values in the lower back and spinal compression forces (Kermavnar et al., 2021). Some applications might suffice to use a passive over an active system to reduce development time, weight, and system complexity, to name just a few aspects. The decision on which type of actuation should be used must be based on technical feasibility and the context of use. Aside from the most frequently used electric motors, there are many alternatives in research. Those range from conventional pneumatic actuators to rather unconventional solutions, like shape memory alloys, carbon nanotubes, or magnetorheological fluid (Manna and Dubey, 2018; Rzyman et al., 2020).

### 3.6. Control modules

**Figure 2.**
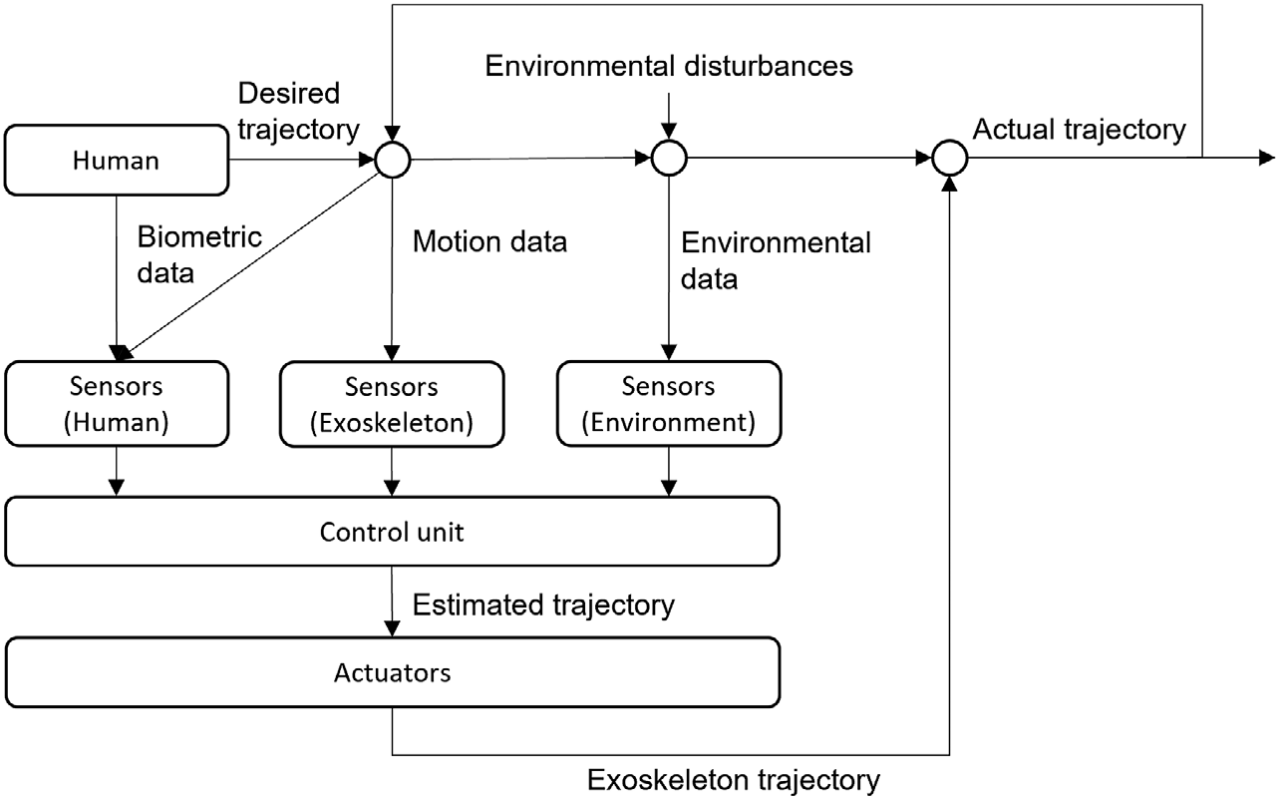
Control schematic of a human-exoskeleton system.

A control algorithm for the actuation is not needed if a system is solely passive. Depending on the task, it might still be necessary to implement sensors to monitor the user, the system, or the environment. Therefore, a cognitive human-machine interface and a fitting interaction design are needed. An appropriate control algorithm is required for an active actuation or a system with both active and passive units. In the literature, there are a lot of proposed control designs and reviews summarizing them. Those reviews are missing categorization, correlation, and evaluation of control strategies regarding the use cases. For example, an exoskeleton that assists in walking needs an entirely different control strategy than an exoskeleton supporting lifting and carrying loads, although methods and algorithms may be adoptable in multiple use cases. New developers are confronted with a variety of literature about different control strategies and are bound to trial and error. Similarly, a wide selection of sensors and measurement methods provide input parameters for those control strategies. A clustered overview of sensors and control designs fitting the use case, kinematics, as well as actuation modules will make the development processes of exoskeletons more efficient. The control system around an exoskeleton is very complex. It includes biometric, environmental, motion, and system inputs that must be computed in real-time. In Figure 2, those are illustrated in a simplistic way. Which biometric, exoskeleton and environmental data are needed strongly depends on the use case (e.g., walking assist or overhead work), supported body parts (e.g., legs or arms) and environment (e.g., medical or industrial). The needed input and output variables have to be defined, and the optimal modalities for transporting data between the human and the exoskeleton robot.

For the modeling of the control, relevant requirements from the kinematic catalog are characteristics like material properties, spring stiffness, restricted and actuated degrees of freedom, or size of movement spaces. Those human- and task-specific parameters lead to requirements for the design of the control variables. Examples of human-specific parameters are the anthropometric dimensions of the individual human body, to which the mechanical structure of the exoskeleton needs to be adapted, and results in changes, for example, dimensions of levers and inertia. Furthermore, task-specific parameters need to be adjustable, like the desired level of assistance or the load weight. This requires a human-machine interface, where those variables can be defined by the users and included in the control algorithm. Considering the interaction modalities increases the efficiency of human-machine interaction. For different modalities for input (speech, motion, touch) and output of data (sound, light/visualization, tactile, haptic, kinesthetic), the context of use and the urgency need to be considered. Further, overstimulating individual senses or distractions can reduce user performance (Wickens, 2002). The user requirements for feedback and the use of multiple modalities must be considered.

The desired trajectories and loads result in the required force and energy output of the exoskeleton. The command variable depends on these values and thus depends on dynamic input variables and the correct detection of disturbance variables. Therefore, accurate real-time monitoring of the state of the human, the machine, and the environment is advantageous. For reliable detection, redundant data acquisition is advised. For each variable that needs to be detected, a catalog of available sensors needs to be put together and the synergies identified. Those vary depending on the design of the kinematics, the intended use case, and the desired supported motion. For human behavior detection, a wide range of sensors is available and in research, for example, EMG, EEG, FSR, goniometer, piezo, ultrasound, infrared, light, and motion-sensors such as IMU and cameras. Since the exoskeleton is in close interaction with the human body, the combined usage of the data for monitoring the system status of the exoskeleton is possible, for example, encoders, energy consumption, and gyroscopes. Especially for events like collisions, the environment also needs to be monitored. Obstacle detection and avoidance, as well as object recognition in load handling, are also possible scenarios where environmental data is necessary for a better control algorithm design.

In literature, the most frequently used control strategy is a closed-loop strategy with the human in the loop. When looking into control techniques, multiple approaches are found in the literature, for example, incorporating some form of PID or impedance control (La Tejera et al., 2021). Those vary depending on the field they are used in (occupational, medical, and military), as well as the intended use case, type of kinematics, and used actuators. For example, when using soft or elastic materials, nonlinear behaviors occur (Xiloyannis et al., 2017). When designing algorithms for the controls, the field of machine learning with the use of neural networks, or more generally artificial intelligence, is very promising. Using them for active exoskeletons means that they can adapt faster to individual human movements and detect the user’s intentions.

Just like kinematics, the control of an active exoskeleton can be evaluated with DHM without the need for a physical prototype. It is especially helpful to identify fitting curves for velocities, accelerations, and forces as a function of the angles of the human joints. The type of control algorithm that is used has a significant influence on the forces exerted on the human body. Since muscle forces and the resulting assistance level needed by the exoskeleton continuously change during a movement, the control design needs to fit those changes to avoid excessive and insufficient support of the human body parts. Khamar et al. (2019) provide examples of such simulation-based control designs.

### 3.7. Evaluation of the framework through an exemplary system

This evaluation concludes the last step within the framework development process. At this point, the framework should be equipped with all necessary solutions and tools to design an exoskeleton. In this step, an exemplary system is developed with the help of the framework in order to evaluate the solutions within and the methodologies behind it. Before building a prototype, a numerical evaluation should be conducted to ensure the effectiveness and usefulness of the exoskeleton. Similar to Section 3.3, with a numerical evaluation based on biomechanical modeling and co-simulation of the soft tissues, the forces acting on and within the human body need to be evaluated to ensure the reduction of strain in the desired body parts. This time, the forces of the motors acting on the body and the behavior of the control algorithm should be included in the assessment and optimized with the simulation if needed. When the control optimization with the simulation is successful, a prototype is needed to verify the simulation results in a subject study. It is crucial to ensure the safe use of the prototype for the test subjects. Similarly to Section 3.4, an experimental evaluation of the (active) prototype needs to be conducted to assess the usability and again verify if the exoskeleton reduces the strain on the body. This can be done by analyzing, for example, the metabolic cost, individual and overall muscle activations, or joint reaction forces (Bae, 2013; Zhou et al., 2017; Knott and Bengler, 2018; Tröster et al., 2018; Gull et al., 2020; Ralfs et al., 2021; Zheng et al., 2021). Furthermore, the comfort level can be estimated by analyzing the forces acting directly on the user (Shao et al., 2014; Shourijeh et al., 2017). Lastly, only field studies and long-term studies will show the exoskeletons’ effects on the users – the reduction of musculoskeletal diseases and avoidance of non-intended use. A standardized process must be derived from such studies to prove the usefulness and safety of the systems introduced to the market, even though it might be necessary to establish research or industrial institutions to carry out such testing to make complex testing possible.

The evaluation of exoskeletons is a well-researched topic, as seen in recent publications about projects such as those published in Germany like Ralfs et al. (2021). There are many standardized methods to choose from for future designers. Not much research is necessary; instead, it needs to be adapted to the cluster of the steps within the framework and matched with the use case, kinematics and actuation. This concludes the last step of the framework and its integrated exoskeleton design process.

### 3.8. Framework for the human-centered development of exoskeletons

**Figure 3.**
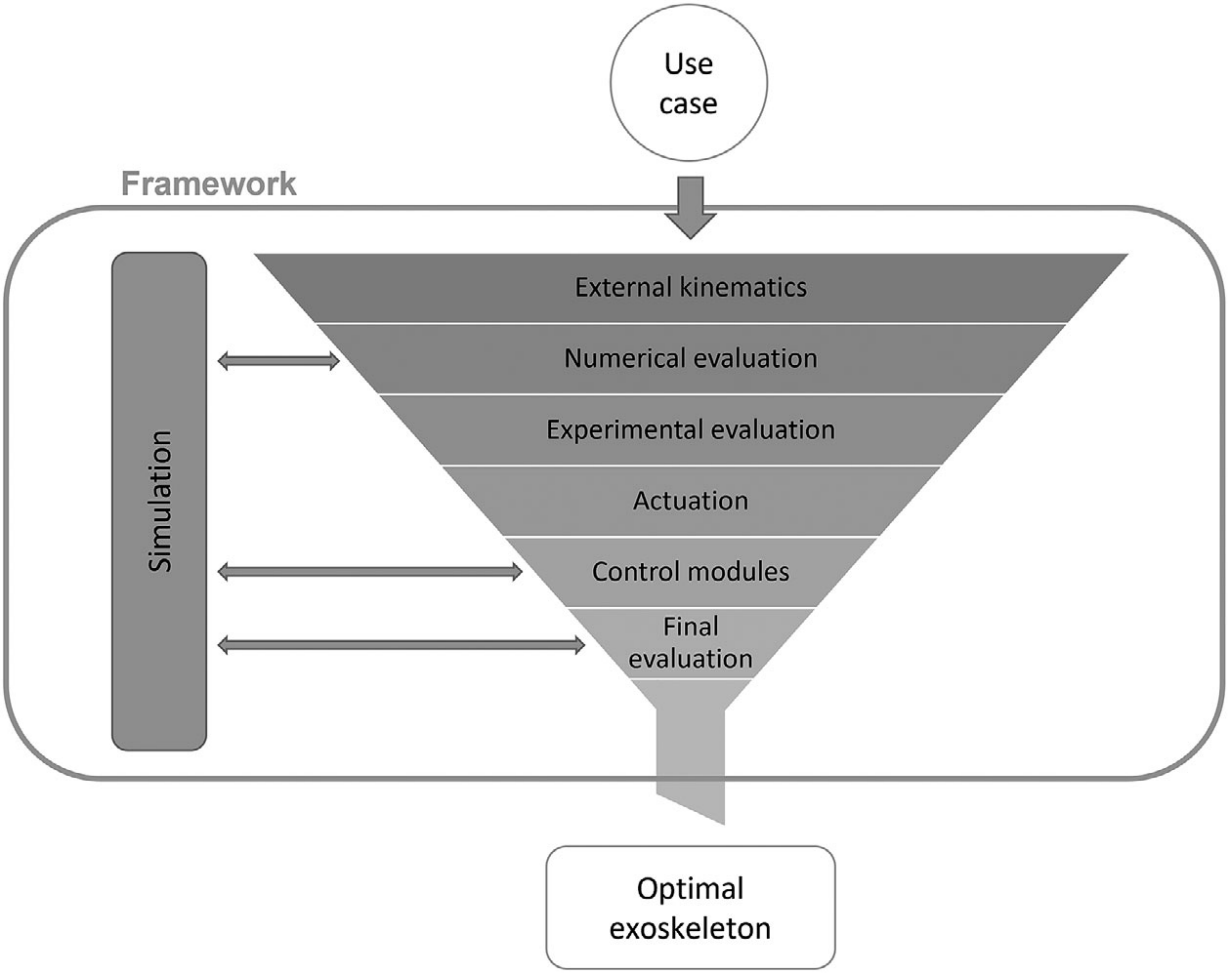
Structure and functionality of the proposed framework for the exoskeleton designer.

The last sections presented a process to generate solutions for a framework with all the necessary steps to design a user- and use-case-centered exoskeleton. The last challenge to make this framework accessible to future exoskeleton developers is to bring tools (simulation and evaluation) and feasible solutions of the system components (kinematics, actuators, control) for exoskeleton design together in a practical, efficient, and effective format. The kinematics, movements, actuators, sensors, and the controlling result in a multidimensional matrix can help to filter the proper solutions for defined use cases and thus enable future engineers to design exoskeletons in a user-centered way without excessive research, prototyping, and trial and error. The actual realization of the resulting exoskeleton is still necessary for a user-centered way to ensure the ergonomic principles of product design and the guaranteed safety of the human user. Thus, long-term and field studies will still be necessary, but the framework will significantly reduce the number of required iterations. The exoskeleton designers will use the framework as pictured in Figure 3. This process is similar to the generation of the framework described in Section 3. However, the users of the framework ideally do not need to expand the framework. The input into the framework is the use case describing the user, the tasks, and the context of usage of the exoskeleton. In the first step, the fitting exoskeleton kinematics are selected from the kinematics generated in the development process of this framework in Section 3.2. These kinematics are then tested through numerical and experimental evaluation. At this point, the best-fitting kinematics is found. The designer then looks up fitting actuators for the use case and the kinematics in the framework. For these actuators, fitting control strategies are identified through simulation and the framework. The prototype of the iteration is now ready to be produced. The prototype will be used in the final evaluation using simulation and user studies. Depending on the outcome, the product development is then finished or needs to be reiterated.

## 4. Conclusion

This article proposes a development process for a framework that will help design exoskeletons in a user- and application-centered way. A lot of fundamental research is still needed to achieve such a framework, and with the proposed process, that framework can be completed with the help of coordinated research projects. The framework is not supposed to be fixed after its first publication but extended by future exoskeleton research and development. The foundation is a platform where this framework is publicized, discussed, and equally complemented with experiences from research and industry.

Exoskeletons have huge potential, but they need to be developed in a structured human-centered process to be useful and accepted by the users. The implications that can be drawn from this rather elaborate and general framework for exoskeleton design are that exoskeletons are potentially individualized solutions, and the mass adaptation of single systems might not be expected, instead of modular solutions. On the other hand, it implies that a lot of fundamental research still needs to be done to understand how a human and a machine can collaborate in such close interaction.

The evaluation process can be used in addition to the framework for the development to test existing systems and enable a standardized comparison between different approaches even though both need to be researched and established in the community.

## Data Availability

Data sharing is not applicable to this article as no new data were created or analyzed in this study.

## Abbreviations

DHM: digital human modeling
FEM: finite-element method
